# Digital gene expression tag profiling of bat digits provides robust candidates contributing to wing formation

**DOI:** 10.1186/1471-2164-11-619

**Published:** 2010-11-06

**Authors:** Zhe Wang, Dong Dong, Binghua Ru, Rebecca L Young, Naijian Han, Tingting Guo, Shuyi Zhang

**Affiliations:** 1 Institute of Molecular Ecology and Evolution, iAIR, East China Normal University, Shanghai 200062, PR China; 2 Yale Systems Biology Institute, Department of Ecology and Evolutionary Biology, Yale University, West Haven, CT 06516, USA; 3 Donnelly Centre for Cellular and Biomolecular Research, University of Toronto, 160 College Street, Toronto, ON, M5S 3E1, Canada; 4 Institute of Zoology, Chinese Academy of Sciences, Beijing 100101, PR China

## Abstract

**Background:**

As the only truly flying mammals, bats use their unique wing - consisting of four elongated digits (digits II-V) connected by membranes - to power their flight. In addition to the elongated digits II-V, the forelimb contains one shorter digit (digit I) that is morphologically similar to the hindlimb digits. Here, we capitalized on the morphological variation among the bat forelimb digits to investigate the molecular mechanisms underlying digit elongation and wing formation. Using next generation sequencing technology, we performed digital gene expression tag profiling (DGE-tag profiling) of developing digits in a pooled sample of two *Myotis ricketti *and validated our sequencing results using real-time quantitative PCR (RT-qPCR) of gene expression in the developing digits of two *Hipposideros armiger*.

**Results:**

Among hundreds of genes exhibiting significant differences in expression between the short and long digits, we highlight 14 genes most related to digit elongation. These genes include two *Tbx *genes (*Tbx3 *and *Tbx15*), five BMP pathway genes (*Bmp3*, *RGMB*, *Smad1*, *Smad4 *and *Nog*), four Homeobox genes (*Hoxd8*, *Hoxd9*, *Hoxa1 *and *Satb1*), and three other genes (*Twist1*, *Tmeff2 *and *Enpp2*) related to digit malformations or cell proliferation. In addition, our results suggest that *Tbx4 *and *Pitx2 *contribute to the morphological similarity and five genes (*Acta1*, *Tnnc2*, *Atp2a1*, *Hrc *and *Myoz1*) contribute to the functional similarity between the thumb and hindlimb digits.

**Conclusions:**

Results of this study not only implicate many developmental genes as robust candidates underlying digit elongation and wing formation in bats, but also provide a better understanding of the genes involved in autopodial development in general.

## Background

**Figure 1:**
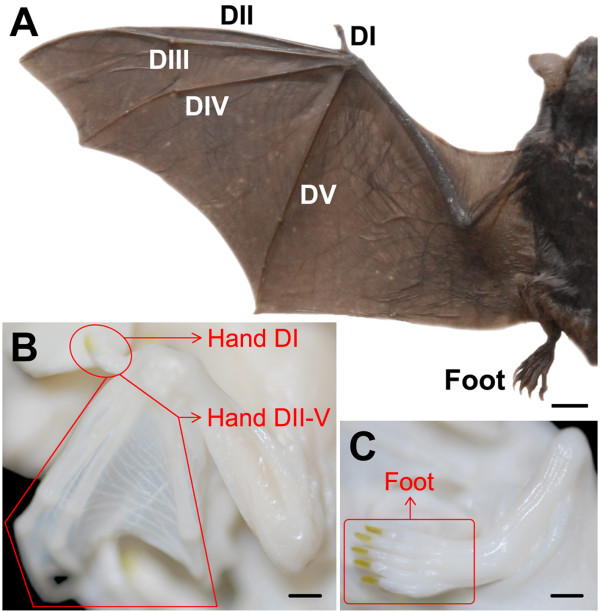
**Limbs of the adult and fetal bats (modified from our previous study **[18]). (A) Left limbs of adult *Myotis ricketti*. DI, DII, DIII, DIV and DV represent digits I-V of the forelimb; (B, C) Left limbs of *Miniopterus schreibersii fuliginosus *in the Fetal Stage as an example of samples used for the *Myotis ricketti *libraries. Libraries Hand DI and Hand DII-V are constructed from forelimb digit I and digits II-V, respectively. Library Foot is constructed from hindlimb digits I-V. Bar = 1 cm in A; bar = 1 mm in B and C.

Bats are a speciose group which constitute approximately 20% of extant mammals, and are the only mammalian group that have powered flight [1]. Unlike the bird wing, made up of an elongated radius and ulna and modified wrist and hand bones that support the flight feathers, bat wings are primarily shaped by elongated digits (digits II-V) that support the wing membranes or dactylopatagia (Figure 1A) [2]. This digit elongation is extreme. For their body length, bats have the longest relative digit lengths with the longest digit (digit III) exceeding the length of the body. However, not all of the forelimb digits are elongated, digit I - the thumb - maintains a short morphology similar to the hindlimb digits and is used to grasp surface or move on the ground (Figure 1). This unique morphology of the bat forelimb digits has sparked interest and motivated investigation by many developmental and evolutionary biologists. Despite these efforts, the mechanisms underlying development of these two distinct digit morphologies (i.e., short and long) of the bat hand remains unresolved. In this study, we capitalize on variation in digit development morphology within the forelimb and between the fore- and hindlimb in bats to identify candidate genes associated with development of distinct digit morphologies. Using DGE-tag profiling (described below) of the thumb, the elongated forelimb digits and the hindlimb digits we identified 14 candidate genes likely associated with digit elongation and seven candidate genes likely associated with the morphological and functional similarity between the thumb and hindlimb digits.

Elongation of the posterior digits (digits II-V) in the bat forelimb is initiated at an early stage when digit anlagen initially appear, embryonic Stage 15 [3]. By embryonic Stage 17, the elongation becomes obvious with lengthening of forelimb digits II-V greatly exceeding that of the thumb and hindlimb digits [3,4]. This rapid elongation of the posterior forelimb digits is thought to results from modification in expression of key developmental genes [5,6]. In fact, several experimental and empirical studies have found differences in expression of limb and digit associated genes between mouse and bat autopodia. First, a mouse transgenic experiment that replaced an enhancer of the mouse *Prx1 *- a transcription factor that regulates long bone growth in mouse - with the orthologous putative enhancer sequence from a bat resulted first in an increase in *Prx1 *expression and second in a small, but significant elongation of the mouse forelimb digits [7,8]. Second, *Shh*, a key factor in limb development, exhibits a unique second phase of signaling late in development of the bat wing that is not present in the development mouse autopodia [9]. Next, other developmental genes including *Fgf8 *and *Bmp2/4 *are up regulated in bat forelimb digits compared to bat hindlimb or mouse digits [10,11]. Finally, the distribution *Hoxd13 *expression is more posteriorly restricted in the bat forelimb relative to mouse fore- and hindlimbs and bat hindlimbs during late embryonic stages [12,13]. While these results indicate that differences in expression of *Hoxd13*, *Fgf8*, *Bmp2/4 *and *Prx1 *during forelimb digit development may contribute to bat digit elongation, the complex developmental changes required to generate the bat wing likely require numerous molecular changes [6]. Thus, further examinations of gene expression during development of the bat digits are critical to identify the genes associated with digit elongation in the posterior forelimb digits and those responsible for the morphological and functional similarities between the short thumb and hindlimb digits.

Here, using a DGE-tag profiling approach, we compared patterns of gene expression among three cDNA libraries formed from distinct regions of the fetal autopodia (the thumb, the four posterior forelimb digits and the all five hindlimb digits) in Rickett's big-footed bat, *Myotis ricketti*. The method of DGE-tag profiling combines the SAGE principle with the massively parallel sequencing technology to study genome-wide gene expression profiles [14]. DGE-tag profiling has three main advantages compared to the conventional microarray technology. First, it is high-throughput sequencing. As a result, data from a single lane of a flow cell (consisting of eight lanes allowing for eight samples) is enough to analyze of genome wide gene expression including weakly expressed genes which cannot be assessed using microarray [15-17]. Next, this type of sequencing data is highly replicable and accurate such that technical replicates have little variation [16]. Finally, sequencing can be performed without *a priori *sequence information, which is required in microarrays [17]. These features of DGE-tag profiling aided our identification of genes associated with digit morphology in bats. Overall, our examination of differentially expressed genes revealed candidate genes associated with both digit elongation as well as morphological and functional similarities between the thumb and hindlimb digits. Finally, our results revealed genes associated with digit position in both limbs.

## Results

### Specimen features

All fetuses obtained were in the "Fetal Stage" which is the last prenatal stage according to bat embryonic staging systems [3]. At this stage, the overall appearance of the embryo is similar to the former stages, but the body size as well as the digit length increases rapidly [3,18]. The genital tubercle had become a vagina or a penis allowing determination of specimen sex. All the specimens used in this study had relatively weak pigmentation indicating an early Fetal Stage as overall body pigmentation increases as fetal development progresses [3,18]. Claws were sharp and keratinized in the thumb and hindlimb digits. Crown rump length (CRL) and forearm length (FA) for both species were recorded in Table 1.

**Table 1:** Specimen id and measurements

Specimen	Captured date	Euthanized date	Gender	CRL (mm)	FA (mm)
Bat M1	13 Feb 2008	28 May 2008	Male	20.78	10.25
Bat M2	13 Feb 2008	6 June 2008	Female	22.39	11.32
Bat H1	24 April 2009	20 May 2009	Male	18.31	32.94
Bat H2	24 April 2009	20 May 2009	Female	19.15	33.87

Bat M1 and M2 are fetal *M. ricketti *used in DGE-tag profiling; Bat H1 and H2 are fetal *H. armiger *used in RT-qPCR. CRL, crown rump length; FA, forearm length.

### Statistics of tag sequencing

**Figure 2:**
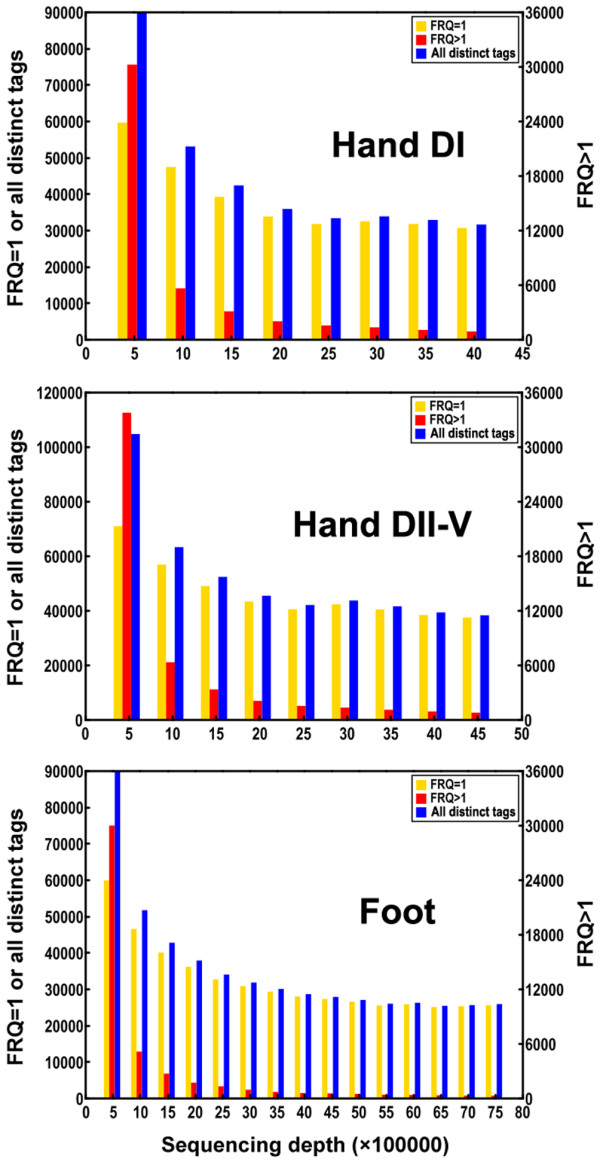
**Saturation measured as the number of new distinct tags added with increasing sequencing depth in each library Hand DI, Hand DII-V and Foot**. FRQ indicates the frequency of distinct tags in the library.

In total, 4377261, 4650734 and 7514943 reads (not including the adapter tags) were sequenced from Library Hand DI, Library Hand DII-V and Library Foot, respectively. Library Hand DI contains 375701 distinct tags, Library Hand DII-V contains 483043, and Library Foot contains 532850. These distinct tags and their genomic frequency as well as the raw data were deposited in NCBI Gene Expression Omnibus (GEO) database with the accession number [GEO: GSE20038]. Most of the distinct tags (over 62%) appeared only once (frequency = 1); however, a small number of distinct tags (less than 1.7%) with frequency higher than 100 made up over 63% of all the tags in all three libraries (Additional file 1, Table S1). When sequencing depths reach 1,000,000 total tags, the number of distinct tags discovered (especially those with frequency >1) drops dramatically in all the three libraries (Figure 2). From that point, increasing sequence depth results in a slow and stable accumulation of new distinct tags indicating that the sequencing has reached saturation (Figure 2).

**Figure 3:**
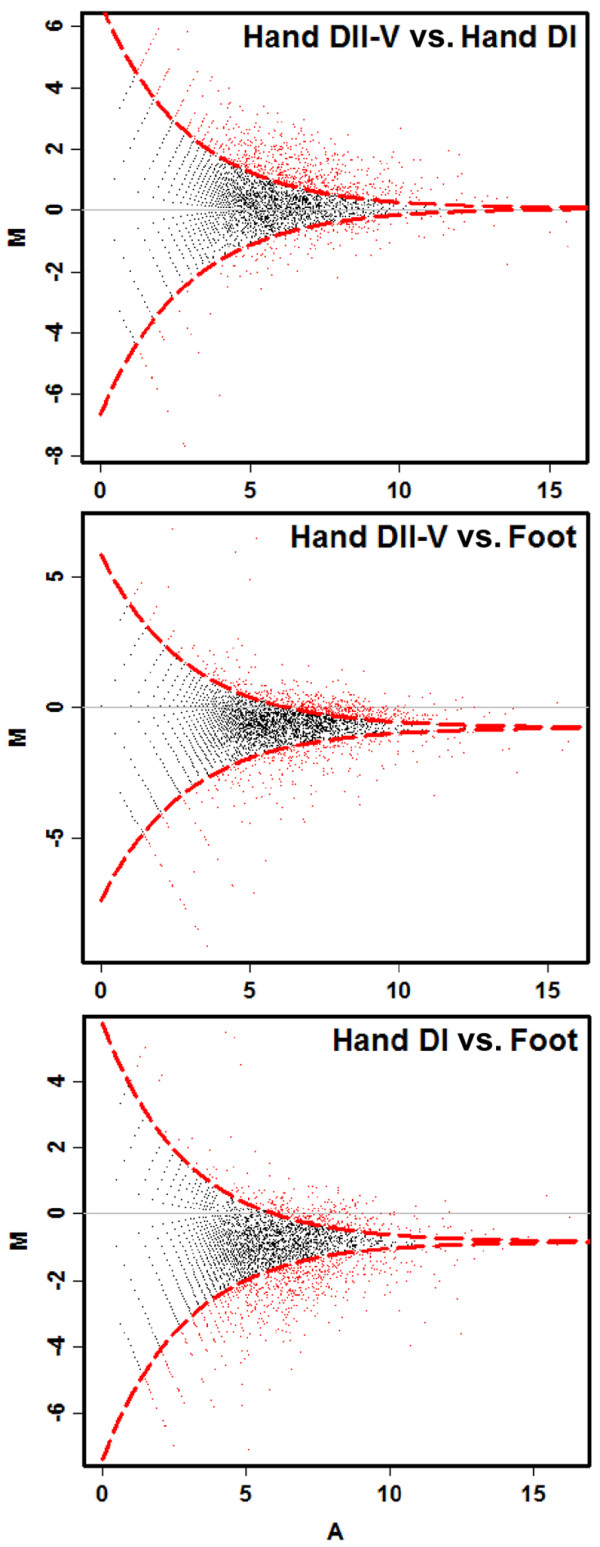
**Pairwise comparison of distinct tag expression between all three libraries (Hand DI, Hand DII-V and Foot) using DEGseq software**. Genes exhibiting significant differences in expression are provided in red (p < 0.001), genes with similar expression in the compared libraries are shown in black.

Differences in expression of genes among the three libraries are indicated in Figure 3. In total, 7080, 8205 and 8967 human genes and 4742, 5666 and 6379 mouse genes were mapped using the tags from the three libraries Hand DI, Hand DII-V and Foot. In total, 38.5% human genes (10490 genes) and 24.6% mouse genes (7843 genes) were mapped using the tags from the three libraries (Additional file 2, Table S2 and additional file 3, Table S3). Some tags mapped to human genes but not mouse genes or vice versa.

### DGE-tag profiling of marker gene expression

The expression patterns of five marker genes (*Tbx5*, *Pitx1*, *Meis2*, *Hoxd10 *and *Krt17*) in the three DGE-tag profiling libraries are consistent with previously reported patterns of expression at embryonic earlier stages (reviewed in the Discussion; Table 2). We found that expression of *Tbx5 *was significantly higher in forelimb digits than the hindlimb digits (p < 0.001) and *Pitx1 *expression was significantly lower in forelimb than in hindlimb digits (p < 0.001). Similarly, as expected, we found that expression of *Meis2 *and *Hoxd10 *were significantly higher in Library Hand DII-V relative to the other two libraries (p < 0.001) and *Krt17 *was significantly lower in Library Hand DII-V relative to the other two libraries (p < 0.001).

**Table 2:** DEGseq analysis of marker genes as well as those highlighted in this study related to digit development

Genes	Tag raw counts			Hand DII-V vs. Hand DI		Hand DII-V vs. Foot		Hand DI vs. Foot	
	
	Hand DII-V	Hand DI	Foot	log2(Fold_change) normalized	p-value	log2(Fold_change) normalized	p-value	log2(Fold_change) normalized	p-value
**Marker genes**									
*Tbx5*	175	134	6*	0.326	0.048	5.6385	1.04E-57	5.3125	1.81E-45
*Pitx1*	6	9	354*	-0.6441	0.3921	-5.1104	4.08E-67	-4.4663	3.04E-63
*Meis2*^*b*^	73*	12	18	2.5457	1.13E-11	2.7922	5.00E-17	0.2465	0.6365
*Hoxd10*^*b*^	61*	3	10	4.2866	1.09E-14	3.3811	5.17E-17	-0.9056	0.289
*Krt17*^*ab*^	68*	267	455	-2.3284	2.28E-40	-1.9474	4.40E-38	0.381	0.0003
**Elongated digits**									
*Tbx3*^*b*^	118*	21	39	2.4312	4.16E-17	2.3695	2.10E-22	-0.0617	0.8689
*Tbx15*^*b*^	171*	41	89	2.0011	2.79E-19	1.7144	2.10E-21	-0.2868	0.2648
*Bmp3*^*b*^	19*	2	5	3.1888	1.04E-04	2.6983	2.64E-05	-0.4905	0.6626
*Rgmb*^*b*^	727*	133	289	2.3914	5.76E-95	2.1031	8.28E-112	-0.2882	0.0435
*Smad1*	264*	122	250	1.0545	4.34E-12	0.8509	1.01E-11	-0.2036	0.1782
*Smad4*	412*	244	351	0.6966	1.19E-09	1.0034	1.60E-22	0.3068	0.0086
*Nog*^*a*^	5**	17	44	-2.1207	1.53E-03	-2.3427	6.61E-06	-0.2219	0.5543
*Hoxd8*	19*	1	4	4.1888	1.81E-05	3.0202	8.64E-06	-1.1686	0.4084
*Hoxd9*	45*	10	13	2.1108	1.88E-06	2.5637	3.17E-10	0.4529	0.4442
*Satb1*	397*	117	286	1.7035	1.48E-34	1.2454	3.85E-30	-0.4581	0.0019
*Hoxa1*^*b*^	61*	14	12	2.0642	4.50E-08	3.118	5.91E-16	1.0538	0.0577
*Twist1*^*b*^	2521*	809	1767	1.5806	7.26E-189	1.285	4.31E-191	-0.2957	3.08E-07
*Tmeff2*^*b*^	95*	22	50	2.0513	1.06E-11	1.6983	2.01E-12	-0.353	0.3091
*Enpp2*^*b*^	27*	0	6	5.6957	8.71E-08	2.9422	1.64E-07	-2.7535	0.0698
**Thumb **&**Foot**									
*Tbx4*^*ab*^	20*	102	390	-2.7057	3.65E-19	-3.4906	2.48E-60	-0.7849	1.61E-08
*Pitx2*	21*	66	127	-1.7112	3.08E-07	-1.8241	6.16E-11	-0.1129	0.589
*Acta1*^*ab*^	412*	1463	4918	-2.1834	1.81E-197	-2.7825	0	-0.5991	9.29E-57
*Tnnc2*^*b*^	115*	534	1544	-2.2744	5.62E-69	-2.9747	6.36E-204	-0.7003	4.41E-26
*Atp2a1*	17*	103	223	-2.6582	3.49E-17	-2.9412	1.12E-30	-0.283	0.0813
*Hrc*	9*	40	67	-2.2112	2.34E-06	-2.1239	1.52E-07	0.0873	0.7535
*Myoz1*	8*	34	58	-2.1466	1.93E-05	-2.0857	1.37E-06	0.0609	0.8391

^a^, homologs of mouse genes, all others are the homologs of human genes; ^b^, genes expression patterns validated by RT-qPCR; * indicates gene expression in the library is significantly higher or lower than in other two libraries (*, p < 0.001; **, p < 0.01).

### Differences in gene expression across the libraries

**Figure 4:**
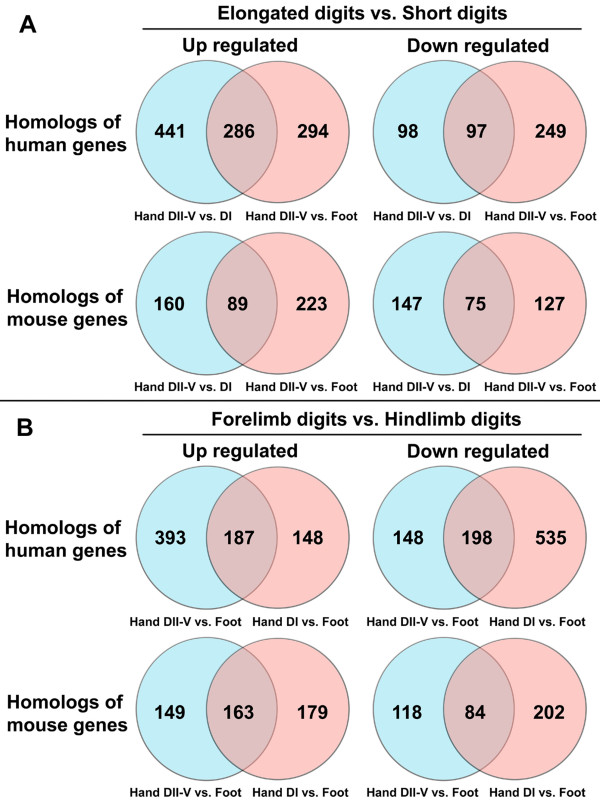
**The number of genes differentially expressed between distinct digit morphologies and digit limb association (p < 0.001)**. (A) Comparison between the elongated wing digits (Library Hand DII-V) and the short digits (Libraries Hand DI and Foot). (B) Comparison between the forelimb digits (Libraries Hand DII-V and Hand DI) and the hindlimb digits (Library Foot).

Additional files 2 and 3 list the results of DEGseq analysis for all the homologs of human (Additional file 2, Table S2) and mouse genes (Additional file 3, Table S3). For the comparison between elongated digits and the short digits, we found that 286 homologs of human genes and 89 homologs of mouse genes were up regulated in Library Hand DII-V compared with other two libraries (p < 0.001, Figure 4) and 97 homologs of human genes and 75 homologs of mouse genes were down regulated in Library Hand DII-V compared with other two libraries (p < 0.001, Figure 4). For the comparison between forelimb digits and the hindlimb digits, we found that 187 homologs of human genes and 163 homologs of mouse genes were down regulated in Library Foot compared with other two libraries (p < 0.001, Figure 4) and 198 homologs of human genes and 84 homologs of mouse genes were up regulated in Library Foot compared with other two libraries (p < 0.001, Figure 4).

Among the differentially expressed genes, we found 14 genes likely related to digit elongation based on results of previous studies (see Discussion). The expression of *Tbx3*, *Tbx15*, *Bmp3*, *Rgmb*, *Smad1*, *Smad4*, *Hoxd8*, *Hoxd9*, *Satb1*, *Hoxa1*, *Twist1*, *Tmeff2 *and *Enpp2 *were significantly higher in Library Hand DII-V compared with other two libraries, and the expression of Nog was significantly lower in Library Hand DII-V (Table 2). Moreover, our results hightlighted 7 genes were likely related to the morphological and functional similarity between the thumb and the hindlimb digits. The levels of *Tbx4*, *Pitx2*, *Acta1*, *Tnnc2*, *Atp2a1*, *Hrc *and *Myoz1 *were significantly higher in Libraries Hand DI and Foot than in Library Hand DII-V (Table 2).

### Real-time quantitative PCR (RT-qPCR)

**Figure 5:**
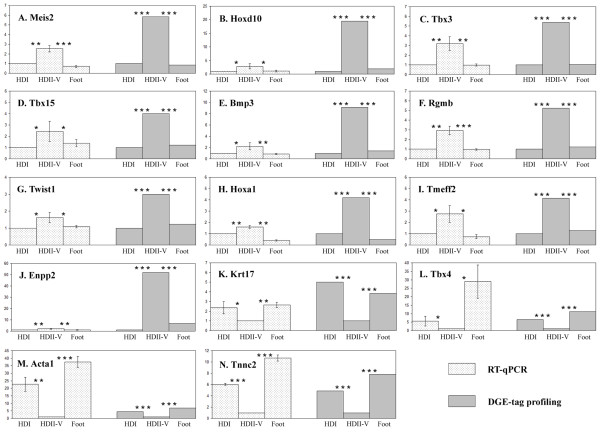
**Patterns of gene expression in hand digit I (HDI), hand digits II-V (HDII-V), and all five foot digits (Foot)**. To validate DGE-tag profiling, expression of 14 genes, exhibiting differences in expression among the *M. ricketti *libraries, were examined in two independent H. armiger embryos using RT-qPCR. (A) Meis2; (B) Hoxd10; (C) Tbx3; (D) Tbx15; (E) Bmp3; (F) Rgmb; (G) Twist1; (H) Hoxa1; (I) Tmeff2; (J) Enpp2; (K) Krt17; (L) Tbx4; (M) Acta1; (N) Tnnc2. The overall pattern and significance of gene expression differences among the samples were concordant using the distinct methods and taxa. The fold changes in expression among samples were reduced in the RT-qPCR in several cases; however this difference is not surprising given the difference in sensitivity of gene detection in the two methods. The y-axis indicates fold change in expression between the samples - HDI, HDII-V, and Foot - using the results from RT-qPCR (dotted bars) and DGE-tag profiling (grey bars) independently. Significance of pairwise comparisons (HDI vs. HDII-V and Foot vs. HDII-V) are indicated by the asterisks between the bars. *, p < 0.05; **, p < 0.01; ***, p < 0.001.

To validate the results of DGE-tag profiling, we performed RT-qPCR of 14 genes which were differentially expressed between libraries (Table 2). All 14 genes exhibited the same overall expression pattern using the distinct methods (Figure 5). *Meis2*, *Hoxd10*, *Tbx3*, *Tbx15*, *Bmp3*, *Rgmb*, *Hoxa1*, *Twist1*, *Tmeff2 *and *Enpp2 *showed significantly higher expression in the posterior forelimb digits (HDII-V) compared with the thumb (HDI) and all five hindlimb digits (Foot). *Krt17*, *Tbx4*, *Acta1 *and *Tnnc2 *showed significantly higher expression in both the thumb (HDI) and all five hindlimb digits (Foot) than in the posterior forelimb digits (HDII-V). The fold change in expression among the samples was often lower in RT-qPCR as compared to DGE-tag profiling; however, this is not surprising given the differences between the two methods in sensitivity of gene detection as well as the fact that the methods were performed in different species.

## Discussion

Massively parallel sequencing is a powerful technology enabling extensive investigation of gene expression profiles. In this study, we obtained over 16.5 million sequences of expressed tags from three bat autopodial libraries, including the thumb (Hand DI), the posterior forelimb digits (Hand DII-V), and all five hindlimb digits (Foot). Using these data, we compared gene expression profiles among the libraries to identify candidate genes associated with elongation of the posterior forelimb digits (Hand DII-V). In addition, we examined gene expression patterns associated with the short digit morphology of the thumb and hindlimb digits as well as gene expression patterns associated with the fore- versus the hindlimb digits. The results of DGE-tag profiling were validated by examining gene expression in a subset of genes using RT-qPCR.

To assess the reliability of our gene expression profiles, we first examined expression of several genes with previously established expression patterns. We compared expression of *Tbx5 *and *Pitx1*, fore- and hindlimb marker genes, in the forelimb and hindlimb digits (Libraries Hand DI and Hand DII-V and Library Foot). Consistent with previous studies, we found that expression of *Tbx5 *was significantly higher in the forelimb digits and *Pitx1 *expression was significantly higher in the hindlimb digits although the embryonic stages used here are much later than those used in previous studies [19,20]. In addition, we compared expression of *Meis2 *and *Hoxd10 *in the fore- and hindlimb digits. Previously published microarray data revealed high levels of expression of these two genes in bat autopodia at Stages 16 and 17 [21]. We found that expression of both genes, *Meis2 *and *Hoxd10*, remained significantly higher in Library Hand DII-V in the Fetal Stage (Table 2). Finally, *Krt17*, a gene expressed in nails and important for the development of nails, exhibited significantly higher expression in the thumb and hindlimb digits [22]. Because only the thumb (Hand DI) and the hindlimb digits have claws, we would expect higher expression of *Krt17 *in the thumb and hindlimb digits relative to the posterior forelimb digits (Hand DII-V) that make up the wing.

In addition to comparing our gene expression profiles to genes with known expression patterns, we further validated our results by performing RT-qPCR using 14 genes which were identified as being differentially expressed between elongated digits and short digits in DGE-tag profiling. With the goal of identifying genes related to wing formation across bat taxa, we collected samples from a different species which belongs to the other suborder of Chiroptera (*H. armiger*). We selected 14 genes likely important for wing formation or for the similarity between the thumb and foot digits (see below). All the 14 genes exhibited the same overall expression pattern in the RT-qPCR as in the DGE-tag profiling (Figure 5). This finding not only provides strong support for the DGE-tag profiling method used in this study, but also maintenance of gene expression patterns across these two phylogenetically distant species provides strong support for the role of these genes in digit elongation in the posterior forelimb digits or morphological and functional similarities between the thumb and hindlimb digits.

Overall, comparisons of gene expression profiles between digit morphologies and limbs identified hundreds of differentially expressed genes. Several interesting patterns have emerged from this data. Specifically, we highlight 21 genes likely related to wing formation or to morphological and functional similarities between thumb and hindlimb digits (Table 2). First, we found 14 genes that are likely associated with digit elongation in bats - two *Tbx *genes (*Tbx3 *and *Tbx15*), five genes from the BMP pathway (*Bmp3*, *Rgmb*, *Smad1*, *Smad4 *and *Nog*), four Homeobox genes (*Hoxd8*, *Hoxd9*, *Satb1 *and *Hoxa1*) and three other gene (*Twist1*, *Tmeff2 *and *Enpp2*) related to either digit malformation or cell proliferation. Next, we identified seven genes (*Tbx4*, *Pitx2*, *Acta1*, *Tnnc2*, *Atp2a1*, *Hrc and Myoz1*) that are likely associated with the morphological and functional similarities between the thumb and hindlimb digits. Expression patterns of these genes in the three distinct digit libraries as well as known function of these genes are described in detail below.

### Genes associated with digit elongation

In general, *Tbx3 *and *Tbx15 *belong to T-box gene family and are involved in skeletogenesis [23,24]. In digit formation specifically, *Tbx3 *is thought to modulate posterior digit identity through Shh and BMP signaling, and *Tbx15 *insufficiency causes Cousin Syndrome which includes congenital dwarfism, moderate brachydactyly (or shortening of the digits) and leg shortening [25-27]. Mutants of *Tbx15 *have reduced bone size and changes in bone shape in the forelimb skeleton indicating an important role in the skeletal development of forelimbs [24].

*Bmp3*, *Rgmb*, *Smad1*, *Smad4 *and *Nog *are all components of the BMP pathway, a pathway critical for bone formation [28]. *Bmp3 *belongs to the transforming growth factor-beta (TGFβ) superfamily and stimulates cell proliferation and differentiation in a concentration dependent fashion [29]. Overexpression of Bmp3 in the chick wing bud has be shown to result in an increase in cell proliferation resulting in lengthening of skeletal elements [30]. *Rgmb*, also named as Dragon, enhances BMP signaling by directly binding to BMP proteins including Bmp2 [31]. Overexpression of Bmp2 and Bmp4 in the developing chick limb leads to a dramatic increase in the volume of cartilage elements [32]. In addition, Bmp2/4 has been shown to be up regulated in the bat forelimb embryonic digits relative to both mouse forelimb digits and bat hindlimb digits [11]. This up-regulation is correlated with increased rate of cartilage proliferation [11]. Beyond up-regulation of BMPs themselves, Smad1 mediates BMP signaling. Specifically, in its phosphorylated state Smad1 forms a complex with Smad4 and then accumulates in the nucleus. This SMAD complex regulates transcription of several target genes [28]. Embryonic manipulation of Smad1 has confirmed that its expression at the phalanx-forming region influences digit identity in the chick limb [33]. In addition, cartilage-specific knockout of *Smad1 *and *Smad4 *reduce chondrocyte proliferation and increase apoptosis resulting in defects of limb elements in the mouse [34,35]. While all the components of the BMP pathway mentioned above stimulate cartilage formation, *Nog *is an antagonist of BMPs and as such inhibits cartilage formation [36]. Mutations of *Nog *disturb the balance of BMP signaling leading to brachydactyly [37]. Thus, our results - exhibiting up regulation of *Bmp3*, *Rgmb*, *Smad1*, *Smad4 *and down regulation of *Nog *in the elongated bat forelimb digits - suggest a role for BMP signaling in the lengthening of the posterior bat forelimb digits.

*Hoxd8*, *Hoxd9*, *Hoxa1 *and *Satb1 *are homeobox genes which specify the anterior-posterior axis and have been implicated as digit identity determining genes [38]. Although *Hoxd8 *and *Hoxd9 *mainly contribute to proximal limb development and are only mildly expressed in the developing digits, we found that *Hoxd8 *and *Hoxd9 *were highly expressed in bat wing digits II-V relative to the thumb and hindlimb digits [39]. Our results also indicate that the homeobox gene *Satb1 *and *Hoxa1 *were highly expressed in bat wing digits II-V relative to the thumb and hindlimb digits. These two genes are highly expressed in cancer cells promoting tumor growth [40,41]. To our knowledge, there are no studies examining expression of *Satb1 *and *Hoxa1 *in limb development making them interesting genes for future investigation.

Similarly to *Satb1 *and *Hoxa1*, *Tmeff2 *and *Enpp2 *are up regulated in carcinomas [42,43]. Previous study showed *Tmeff2 *contributes to cell proliferation. Enpp2 which is expressed in precartilaginous condensations can be strongly activated by Hoxa13 and Hoxd13 which are crucial factors for digit identity and development [44]. Finally, knowledge of the role of *Twist1 *and the digit development comes from pathological studies [45]. Mutations *Twist1 *have been found in patients with Saethre-Chotzen syndrome which is associated with digit malformation [45]. Overexpression of Twist1 inhibits osteoblast differentiation and prevents premature fusion of skeletons [46].

### Genes associated with similarities between the thumb and hindlimb digits

In addition to genes related to bat digit elongation, we found several genes that may contribute to the morphological and functional similarities between the bat thumb and hindlimb digits. First, *Tbx4 *and *Pitx2 *are primarily associated with hindlimb development. *Tbx4 *has also been shown to regulate bone size. Specifically, in the developing mouse forelimb, overexpression of these genes results in shortening of the forelimb skeletons [47,48]. Next, we found several genes that are likely associated with the distinct musculature and function of the short digits. Because the clawed thumb of bats is used to cling to the cave while roosting and the clawed hindlimb digits are used for hanging upside down these digits should be richer in skeletal muscles than the elongated digits. In fact, expression of several skeletal muscle associated genes - *Acta1*, *Tnnc2*, *Atp2a1*, *Hrc *and *Myoz1 *- was significantly higher in the thumb and hindlimb digits then in the elongated posterior forelimb digits [49].

## Conclusions

Using genome-wide sequencing method we have identified many genes differentially expressed in the developing digits of the bat autopodia. Several of these genes are highly related to digit formation and thus likely play important roles in the development of bat wing. Our results provide robust candidate genes for future functional tests and molecular evolution comparison with other mammals to fully understand the mechanisms of wing formation and the evolution of bat flight.

## Methods

### Animal collection and breeding

All procedures were carried out in accordance with the Policy on the Care and Use of Animals, approved by the Ethical Committee, East China Normal University. *Myotis ricketti*, Rickett's big-footed bat, belongs to suborder Yangochiroptera and is widely distributed in China [50]. Their feet - which are used to forage on fishes - are larger than those of other bat species (Figure 1A) [51]. As in many species of bats, copulation in *Myotis ricketti *occurs in autumn and spermatozoa are stored in the female's uterus and oviducts through the winter hibernation. Ovulation and subsequent fertilization begin in the spring with parturition occurring in summer [52]. Because gravid *M. ricketti *could not be found during the summer breeding season, we capitalized on sperm storage by females and captured 10 hibernating female *M. ricketti *from a cave (39°42'N, 115°43'E) in Beijing on February 13, 2008. In addition to *Myotis ricketti*, we captured three gravid *Hipposideros armiger *from a cave (30°20'N, 117°50'E) in Anhui province of China on April 24, 2009 to use for quantitative real-time PCR confirmation sequencing results. *H. armiger*, the great leaf-nosed bat, belongs to the other suborder Yinpterochiroptera and is widely distributed in subtropical and tropical zones of Asia [53].

After capture, bats were kept in a large cage (1.2 × 1 × 1 m) covered with wire netting allowing them to hang. The cage was kept in a dark, temperature controlled room. The temperature was maintained between 18 and 24°C. Water was freely available and meal worms mixed with a powdered multivitamin and calcium tablets were provided daily.

### Specimen processing

*M. ricketti *in the captive colony were euthanized by decapitation on May 28 and June 6, 2008 (Table 1). *H. armiger *were euthanized on May 20, 2009 (Table 1). Fetuses from the gravid females were put into ice cold PBS. The thumb, the remaining forelimb digits (metacarpi, phalanges and dactylopatagium) and all five hindlimb digits (metatarsi and phalages) were dissected from the fetuses (Figure 1B and 1C) and stored in liquid nitrogen until use. All other components of the fetus were fixed overnight in Bouin's fluid, washed with several changes of 70% ethyl alcohol and stored at room temperature. Crown rump length (CRL) and forearm length (FA) were measured for further estimation of the embryonic stages.

### Digital gene expression tag profiling (DGE-tag profiling)

To reduce sex-specific gene expression patterns, we pooled the samples from a male and a female fetus of *M. ricketti *(Bat M1 and Bat M2 respectively, see Table 1). Tag libraries were prepared using the DGE-Tag Profiling for NlaIII Sample Prep kit from Illumina according to the manufacturer's instructions. In brief, total RNA was extracted from forelimb digit I, forelimb digits II-V and all five hindlimb digits using TRIzol^® ^Reagent (Invitrogen, Cat. No. 15596026, US), treated with DNase I (Roche, Cat. No. 04716728001, Germany) and converted to double stranded cDNA using Oligo dT beads. Subsequently, the cDNA samples were digested using the restriction enzyme NlaIII, which recognizes and cuts the most 3' "CATG". Once digested into fragments, cDNA samples were ligated to Illumina specific adapter A, which contains a recognition site for enzyme MmeI. The Illumina specific adapter B was ligated after MmeI digestion (Illumina, Cat. No. FC-102-1005, US). Using this technique, three DGE libraries (Libraries Hand DI, Hand DII-V and Foot) from the three samples (Figure 1B and 1C) were constructed such that each molecule in the library is a 21 bp tag derived from a single transcript with Illumina specific adapters attached to both ends. Next, the 21 bp tags were enriched using PCR primers that anneal to the adaptors and clusters were created on a flow cell using a Solexa Cluster Station following the manufacturer's instructions (Illumina, Cat. No. FC-103-1004, US). Finally, massively parallel sequencing-by-synthesis was performed on the Illumina Genome Analyzer. Image analysis, base calling, extraction of 21 bp tags, and tag counting were performed using the Illumina pipeline. The number of times that a unique tag sequence is detected represents the quantitative expression level of the corresponding transcript in the tissue. The raw data, including tag sequences and counts, were deposited in NCBI Gene Expression Omnibus (GEO) database with the accession number [GEO: GSE20038]

### In silico analysis

We analyzed saturation of each of the three libraries by examining the addition of new distinct tags associated with the increase of sequencing depth (frequency = 1, > 1 and all distinct tags). Because little gene annotation exists for the bat genome, all distinct tags were aligned against the well annotated NCBI human and mouse Refseq cDNA sequences using SOAP2 [54,55]. Taking into account the differences between species, one base pair mismatches were allowed during the mapping of tags to cDNA sequences. Tags that matched to more than one gene were not used in analyzing differential expression among the libraries. The identified cDNA sequences were mapped to NCBI Entrez GeneID. Differential gene expression analysis was performed using DEGseq software [56]. We compared libraries Hand DII-V with Hand DI and Foot to identify differentially expressed genes between elongated digits and short digits. We also compared libraries Hand DII-V and Hand DI with Foot to identify differentially expressed genes between the forelimb and hindlimb digits.

### Real-time quantitative PCR (RT-qPCR)

Total RNA were extracted from forelimb digit I, forelimb digits II-V and all five hindlimb digits of two *H. armiger *(Bat H1 and H2, Table 1), using TRIzol^® ^Reagent (Invitrogen, Cat. No. 15596026, US) and treated with DNase I (Roche, Cat. No. 04716728001, Germany). cDNA synthesis and qPCR was performed using the SYBR^® ^PrimeScript^® ^RT-PCR Kit (TaKaRa, Cat. No. DRR063A, Japan) on a Applied Biosystems 7300 Real-Time PCR System (ABI, US). RT-qPCR for each *H. armiger *(Bat H1 and H2, Table 1) was performed in duplicate. β-actin was taken as a reference gene and it was amplified by a pair of previously published primers [57]. We designed 14 pairs of degenerate primers to amplify 14 target genes (*Meis2*, *Hoxd10*, *Tbx3*, *Tbx15*, *Bmp3*, *Rgmb*, *Twist1*, *Hoxa1*, *Tmeff2*, *Enpp2*, *Krt17*, *Tbx4*, *Acta1 *and *Tnnc2*). PCR products of each gene were ligated into a pMD^®^19-T vector (TaKaRa, Cat. No. D102B, Japan), cloned, and sequenced using the Big Dye Terminator kit on an ABI 3730 DNA sequencer (Applied Biosystems, US). Using the obtained sequences, we designed gene specific primers for each target gene for qPCR. 2^-^ΔΔ^CT ^method was used to quantify gene expression, data are expressed as mean ± SD [58]. Student's t-test was performed to examine the difference of gene expression between two different samples. Sequences of forward and reverse primers for normal PCR and RT-qPCR of each gene are shown in additional file 4, Table S4. The partial CDS of the genes were deposited at GenBank under accession number HM777024 - HM777038.

## Abbreviations

CRL: Crown rump length; FA: forearm length; FRQ: frequency.

## Authors' contributions

ZW participated in design of the study. ZW and DD carried out the data analysis and wrote the manuscript. ZW, BR and TG performed the experiments. RLY contributed to the final manuscript preparation. NH fed and examined the bats. SZ conceived of the study, participated in its design, and supervised the work. All authors read and approved the final manuscript.

## Supplementary Material

Additional file 1 **Table S1.xls**. Tag frequency. The number of distinct and total tags counted at different frequencies in each library. FRQ indicates the frequency of distinct tags in the library. Click here for file

Additional file 2 **Table S2.xls**. Expression and analysis of human gene homologs in bat digits The expression of human gene homologs mapped by tags in each library and the results of DEGseq analysis for the study of gene differential expression [56]. Symbol indicates NCBI official gene symbol; GeneID indicates NCBI Entrez Gene ID. The raw count of each mapped gene is presented under its library name (Libraries Hand DI, Hand DII-V or Foot). All other parameters (log2(fold_change), log2(fold_change) nomalized, z-score, p-value, q-value and signature) were calculated using the DEGseq [56]. Click here for file

Additional file 3 **Table S3.xls**. Expression and analysis of mouse gene homologs in bat digits The expression of mouse gene homologs mapped by tags in each library and the results of DEGseq analysis for the study of gene differential expression [56]. Symbol indicates NCBI official gene symbol; GeneID indicates NCBI Entrez Gene ID. The raw count of each mapped gene is presented under its library name (Libraries Hand DI, Hand DII-V or Foot). All other parameters (log2(fold_change), log2(fold_change) nomalized, z-score, p-value, q-value and signature) were calculated using the DEGseq [56]. Click here for file

Additional file 4 **Table S4.xls**. Sequences of primers used in PCR and RT-qPCR Nucleotide sequences of the primers used in PCR and RT-qPCR of candidate genes underlying digit distinct morphologies (Figure 5). ^a ^indicates previous published primers [57]. The lengths of introns are inferred from the homologs of human genes. Click here for file
